# Supplementary figures and images for: A DNA replication-independent function of pre-replication complex genes during cell invasion in C. elegans
Source: PLoS Biol. 2022 Feb 22;20(2):e3001317. doi: 10.1371/journal.pbio.3001317 (PMC8863262; doi:10.1371/journal.pbio.3001317)

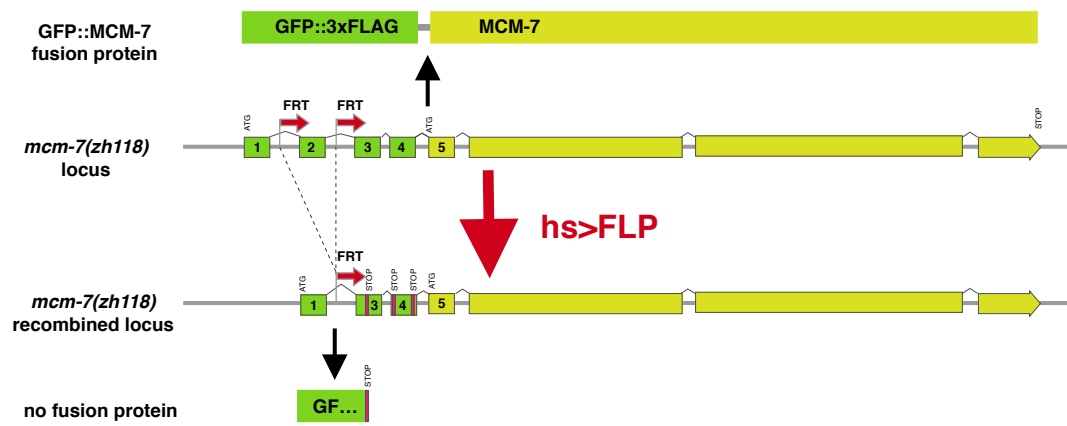

Supplement: S1 Fig — Structure of the frt::gfp::mcm-7(zh118) locus before and after FLP-mediated recombination. The gfp coding regions and protein sequence are labeled in green and the mcm-7 region in yellow. The stop codons generated by the frameshift after the excision of exon 2 are indicated in red. FLP, flippase; FRT, FLP recognition target sequence. (PDF) [file pbio.3001317.s001.pdf]

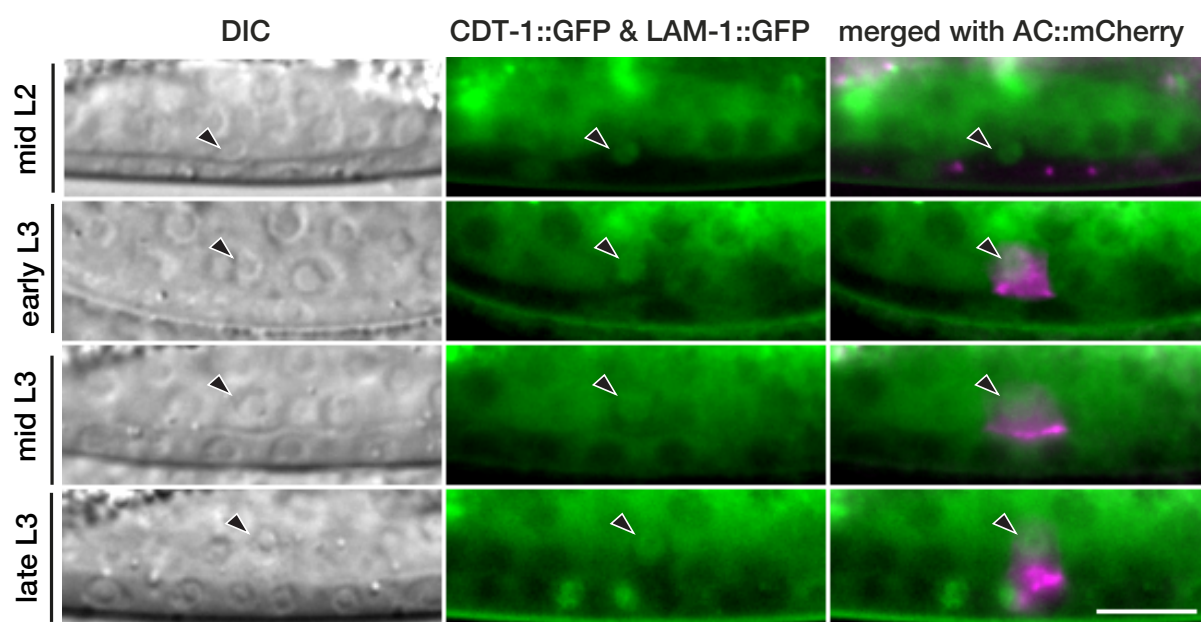

Supplement: S2 Fig — GFP::CDT-1 (zhIs120) and LAM-1::GFP expression beginning at the mid-L2 stage, shortly before AC specification, until the late-L3 (P6.pxx) stage after BM breaching are shown. DIC images are shown in the left panels; CDT-1::GFP and LAM-1::GFP expression in green are shown in the middle panels. The right panels show the GFP images merged with the AC marker ACEL>mCherry (zhIs127) in magenta. The arrowheads point to the AC nuclei. The scale bar is 5 μm. AC, anchor cell; BM, basement membrane; DIC, differential interference contrast. (PDF) [file pbio.3001317.s002.pdf]

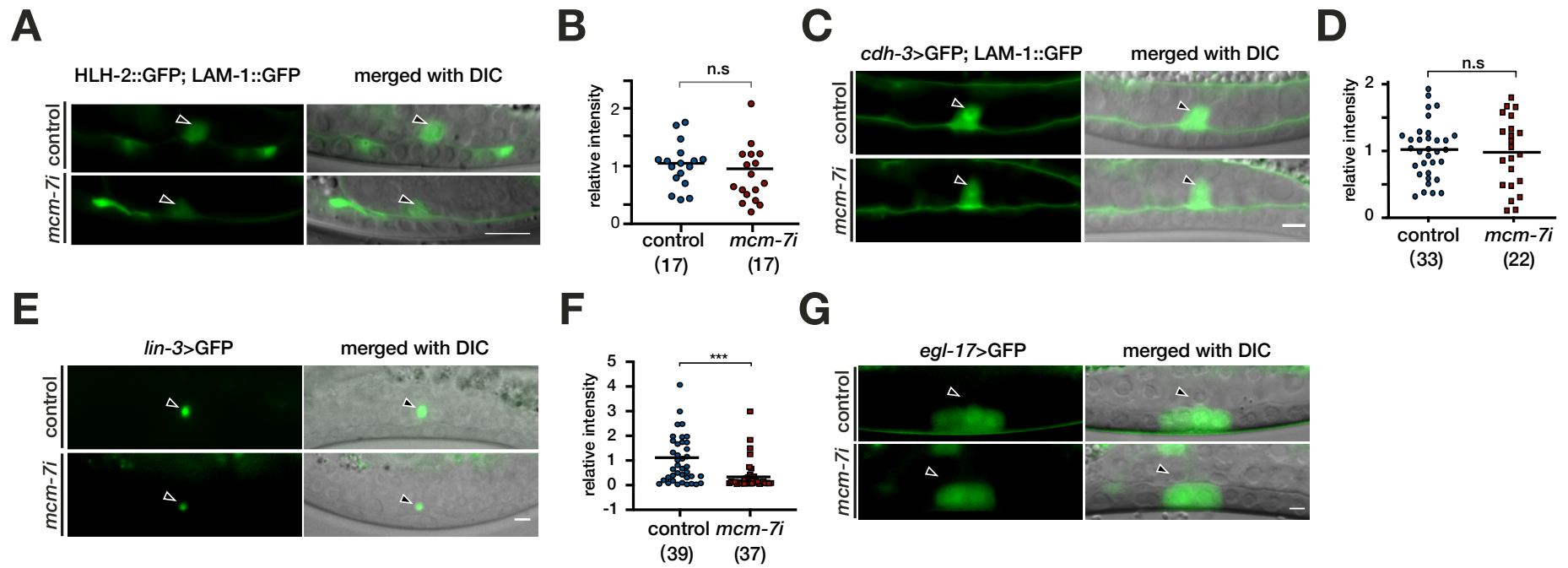

Supplement: S3 Fig — (A) Expression of a translational reporter for the HLH-2 transcription factor together with the LAM-1::GFP BM marker in control animals (top row) and in mcm-7 RNAi-treated animals (bottom row) and (B) quantification of HLH-2 expression levels in the AC. (C) Expression of a transcriptional reporter for the CDH-3 proto-cadherin together with the LAM-1::GFP BM marker in control animals (top row) and in mcm-7 RNAi-treated animals (bottom row) and (D) quantification of cdh-3 expression levels in the AC. (E) Expression of a transcriptional reporter for the EGF homolog lin-3 in control animals (top row) and in mcm-7 RNAi-treated animals (bottom row) and (F) quantification of lin-3 expression levels in the AC. (G) Expression of the 1° fate marker egl-17 in the VPCs of control animals (top row) and in mcm-7 RNAi-treated animals (bottom row). Left panels show the GFP signals and right panels the GFP channel merged with the DIC images. The numbers in brackets in the graphs refer to the numbers of animals analyzed, and the horizontal lines indicate the median values. Statistical significance was determined with a two-tailed t test for independent samples and is indicated as n.s. for p > 0.05 and *** for p < 0.001. See S1 Data for the numerical values used to generate the graphs in (B), (F), and (D). The scale bars are 10 μm in (A) and 5 μm in (C), (E), and (G). AC, anchor cell; DIC, differential interference contrast; n.s., not significant; VPC, vulval precursor cell. (PDF) [file pbio.3001317.s003.pdf]

**A**

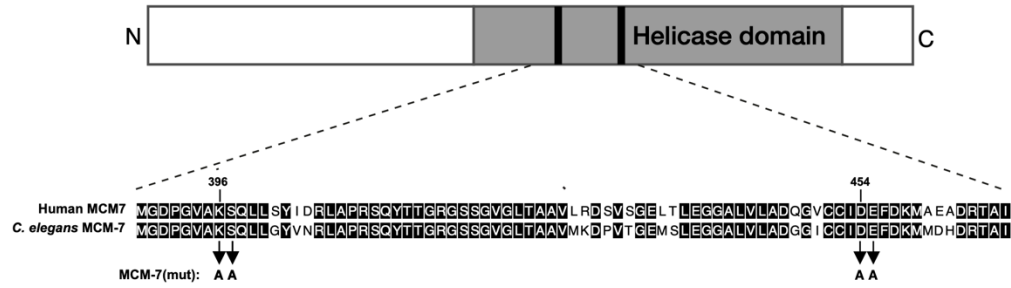

**B**

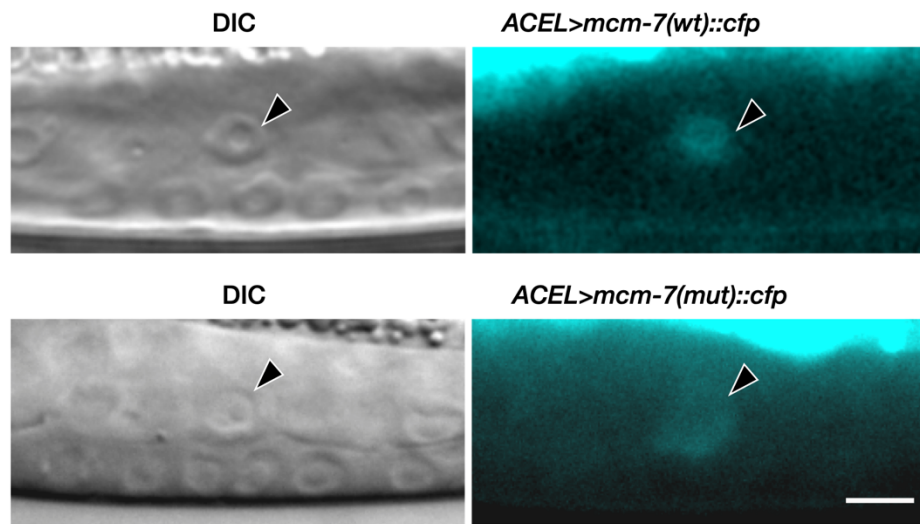

**C**

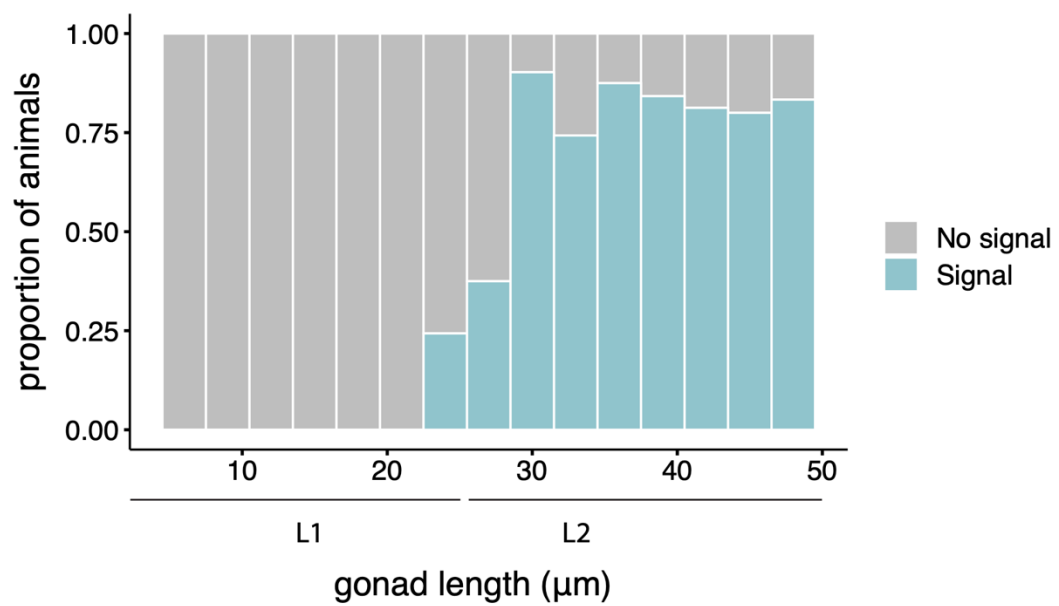

Supplement: S4 Fig — (A) Alignment of the ATPase motif in the helicase domain of human and C. elegans MCM-7. Conserved residues are highlighted in black. The 4 amino acid substitutions eliminating the helicase activity in the ACEL>mcm-7(mut)::cfp transgene are shown. (B) AC-specific expression of the ACEL>MCM-7::CFP wild-type and mutant proteins. The arrowheads in the DIC images point at the AC nuclei. (C) Quantification of ACEL>MCM-7(wt)::CFP expression in the somatic gonad from the L1 to the ate L2 stage. Larval stages were assigned according to the gonad lengths, as described in [76]. The x-axis shows the gonad length measured as distance between the 2 distal tip cells and the y-axis the proportion of animals showing MCM-7(wt)::CFP expression in the AC (n = 391). See S1 Data for the numerical values used to generate the graph. The scale bar in (B) is 5 μm. AC, anchor cell; DIC, differential interference contrast. (PDF) [file pbio.3001317.s004.pdf]

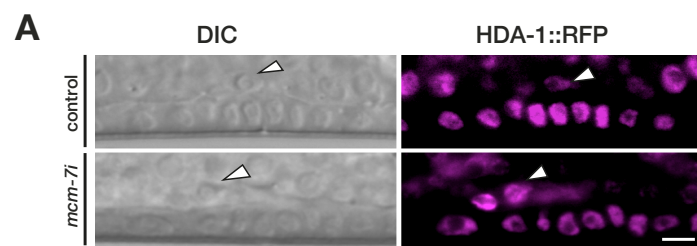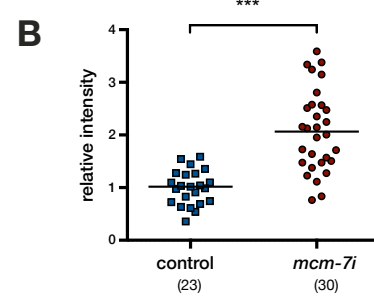

Supplement: S5 Fig — (A) HDA-1::RFP expression in the AC in control RNAi (top row) and mcm-7 RNAi-treated animals (bottom row). The scale bar is 5 μm. (B) Quantification of HDA-1::RFP expression levels in the AC. The right panels show the DIC images and the right panels the fluorescence signal. The arrowheads point at the AC nuclei. The numbers in brackets in the graphs refer to the numbers of animals analyzed, and the horizontal lines indicate the median values. Statistical significance was determined with a two-tailed t test for independent samples and is indicated as *** for p < 0.001. See S1 Data for the numerical values used to generate the graph. AC, anchor cell; DIC, differential interference contrast. (PDF) [file pbio.3001317.s005.pdf]

**A**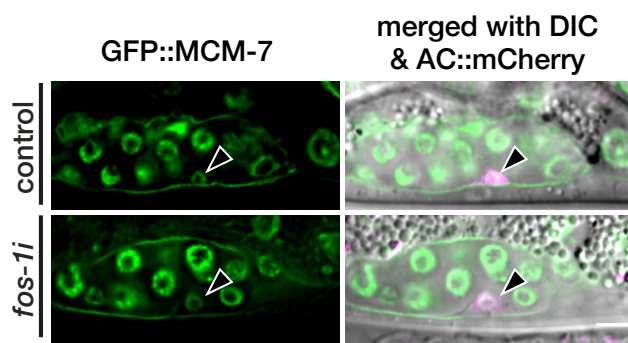**B**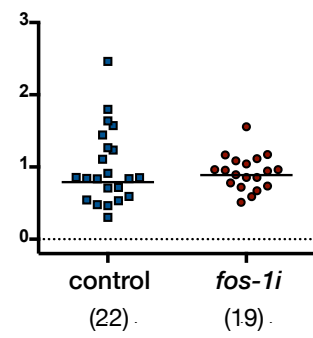

Supplement: S6 Fig — (A) GFP::MCM-7 expression in the AC in control RNAi (top row) and fos-1 RNAi-treated animals (bottom row) The scale bar is 5 μm. (B) Quantification of GFP::MCM-7 expression levels in the AC. The right panels show the DIC images overlaid with the GFP::MCM-7 and the AC::mCherry signals. The numbers in brackets in the graphs refer to the numbers of animals analyzed, and the horizontal lines indicate the median values. See S1 Data for the numerical values used to generate the graph. AC, anchor cell; DIC, differential interference contrast. (PDF) [file pbio.3001317.s006.pdf]

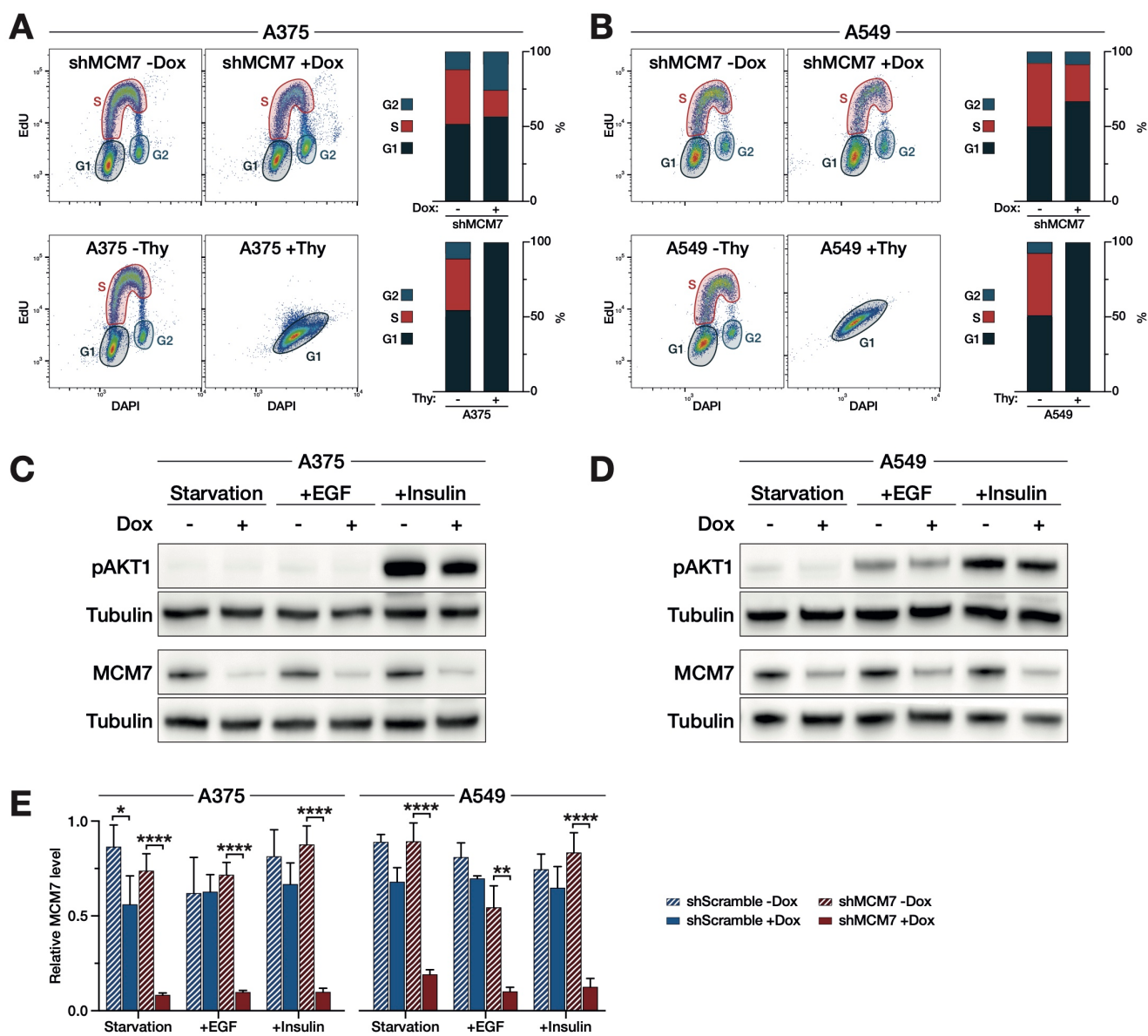

Supplement: S7 Fig — (A) Cell cycle analysis by flow cytometry of A375 and (B) A549 cells expressing shScramble or shMCM7 without or with double thymidine block (+Thy). (C) Western blot analysis of phospho-AKT and MCM7 levels upon MCM7 knock-down in Thy-arrested, serum-starved EGF and insulin-stimulated A375 and (D) A549 cells. The western blots of 1 out of 3 biological replicates for each cell line are shown. See S1 Raw Images for the original images of all western blots. (E) Quantification of MCM7 protein levels in shScramble and shMCM7 expressing A375 and A549 cells. The averaged measurements of 4 biological replicates are shown. Statistical significance in (E) was determined with two-way ANOVA followed by two-tailed t tests for independent samples and is indicated as * for p < 0.05 and as **** for p < 0.0001. See S1 Data for the numerical values used to generate the graph s in (A), (B), and (E). The flow cytometry data can be found in the FlowRepository (https://flowrepository.org/) under accession number FR-FCM-Z4XE. Dox, doxycycline; Thy, thymidine. (PDF) [file pbio.3001317.s007.pdf]

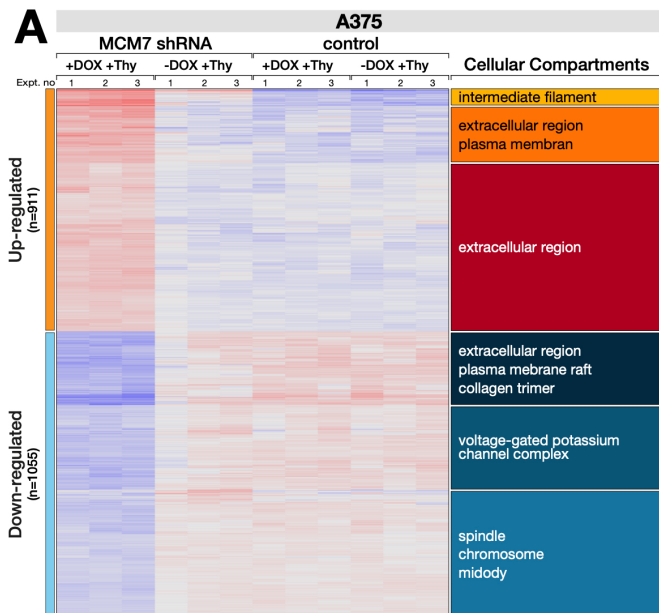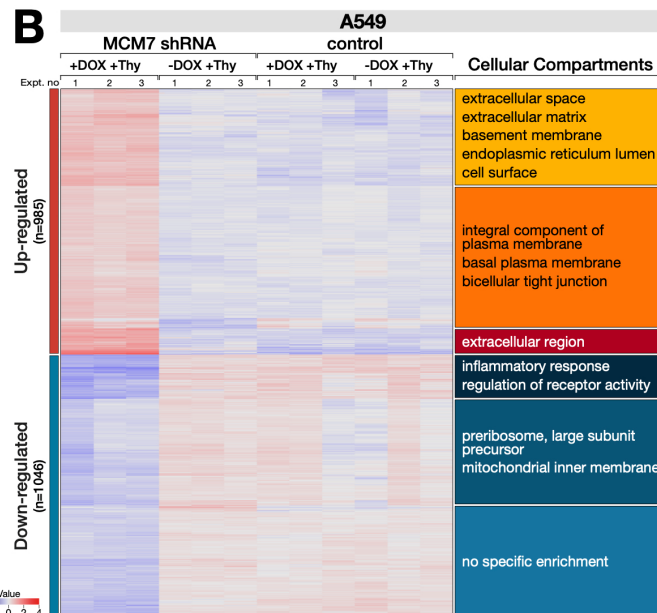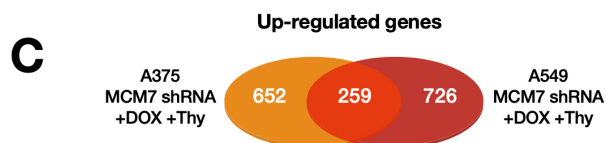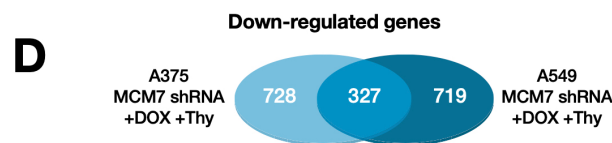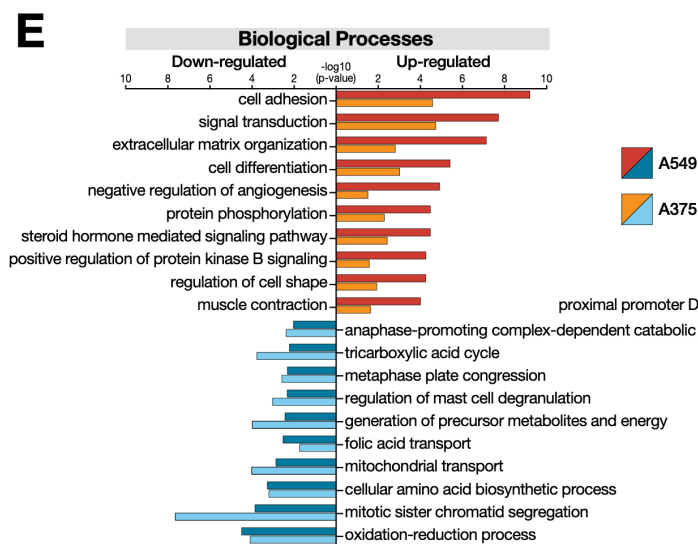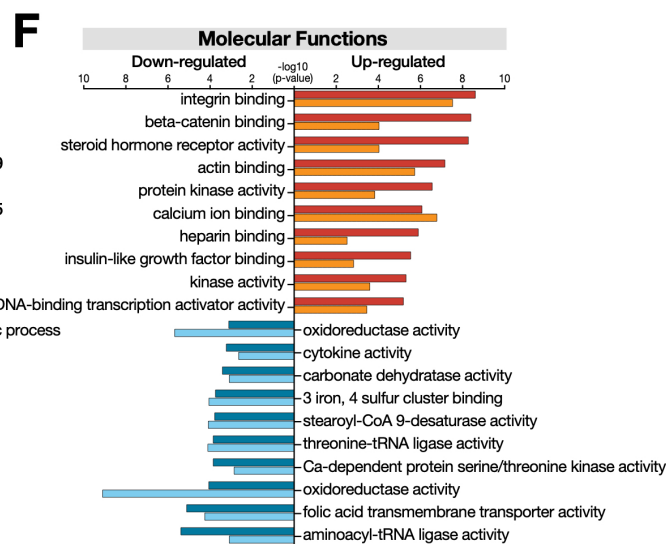

Supplement: S8 Fig — (A) Differentially expressed genes after Dox-inducible MCM7 knock-down in G1/S-arrested (+Thy) A375 cells and (B) A549 cells were identified by RNAseq analysis. For each cell line, the data of 3 independent biological replicates were analyzed. A clustering analysis of up- and down-regulated genes with a >1.5-fold expression change with p < 0.01 and an FDR <0.05 is shown. Control cells were the parental A549 and A375 cell lines grown with or without Dox. For all samples, RNA was extracted from populations, in which at least 95% of the cells had been arrested in the G1/S phase boundary by double Thy block as illustrated in S7 Fig. The colored boxes on the y-axes indicate the identified gene clusters according to the cellular compartments of their gene products. The significantly changed transcripts are listed in S2 Data, and the RNAseq data generated in this study are available at the NCBI GEO (http://www.ncbi.nlm.nih.gov/geo/) under accession number GSE149523. (C) Overlap between the up-regulated and (D) down-regulated genes in the 2 cell lines. The overlap between the MCM7-regulated genes in the 2 cell lines is highly significant (p < 0.001), as the expected number of randomly overlapping genes determined by bootstrap analysis is 42 for the up- and 52 for the down-regulated genes. (E) GO enrichment analysis according to the biological processes and (F) molecular functions of the genes regulated by MCM7 in both cell lines. The red or orange bars indicate the top 10 GO terms enriched in up-regulated genes and the dark or light blue bars the top 10 GO terms enriched in down-regulated genes. The lengths of the bars correspond to the ±log10 of the p-values indicating the significances of enrichment in each cell line. Dox, doxycycline; FDR, false discovery rate; GEO, Gene Expression Omnibus; GO, Gene Ontology; Thy, thymidine. (PDF) [file pbio.3001317.s008.pdf]

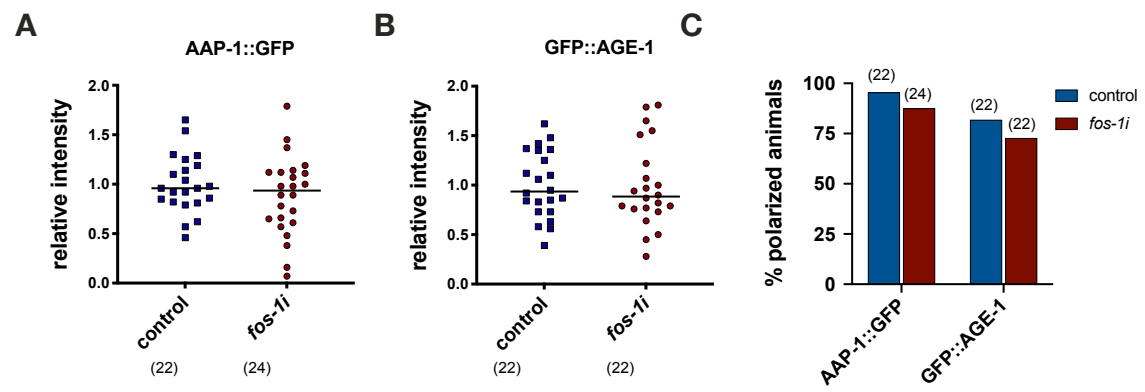

Supplement: S9 Fig — (A) Quantification of AAP-1::GFP and (B) AGE-1::GFP expression levels in the AC of control and fos-1 RNAi-treated animals. (C) Fraction of animals exhibiting polarized AAP-1::GFP and GFP::AGE-1 localization. The numbers of animals scored are indicated in brackets. The horizontal lines indicate the median values. No significant differences were found in two-tailed t tests for independent samples (p > 0.2). See S1 Data for the numerical values used to generate the graphs in (A-C). AC, anchor cell. (PDF) [file pbio.3001317.s009.pdf]

LAM-1::GFP

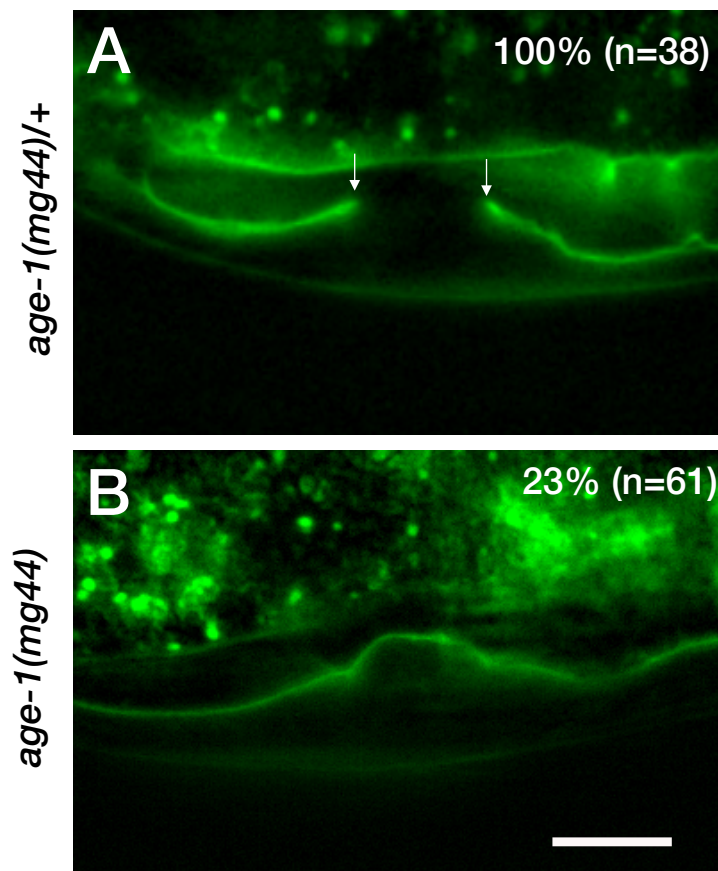

Supplement: S10 Fig — (A) BM breaching visualized with the LAM-1::GFP marker in age-1(mg)/+ heterozygous and (B) homozygous age-1(mg44) L4 larvae in the F2 generation. All heterozygous age-1(mg)/+ siblings carrying the mnC1 balancer showed normal BM breaching (n = 38), while 23% of the F2 generation age-1(mg44) animals (n = 61) exhibited no or only partial BM breaching. BM, basement membrane. (PDF) [file pbio.3001317.s010.pdf]
