# Supplementary material for: A DNA replication-independent function of pre-replication complex genes during cell invasion in C. elegans
Source: PLoS Biol. 2022 Feb 22;20(2):e3001317. doi: 10.1371/journal.pbio.3001317 (PMC8863262; doi:10.1371/journal.pbio.3001317)
Supplement: S1 Raw images — (PDF) [file pbio.3001317.s018.pdf]

Original Westernblots Figure S7C&E

A375 shMCM7

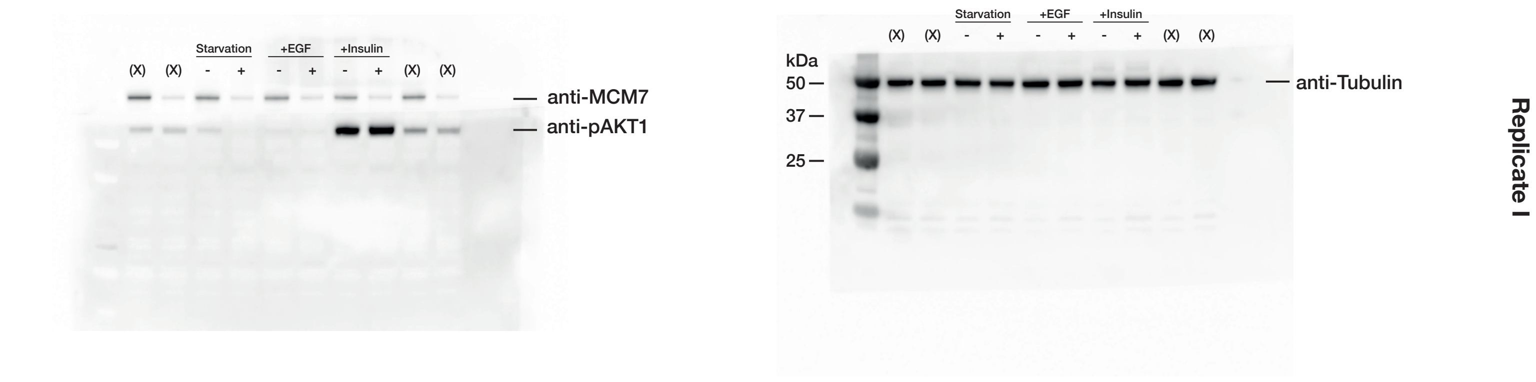

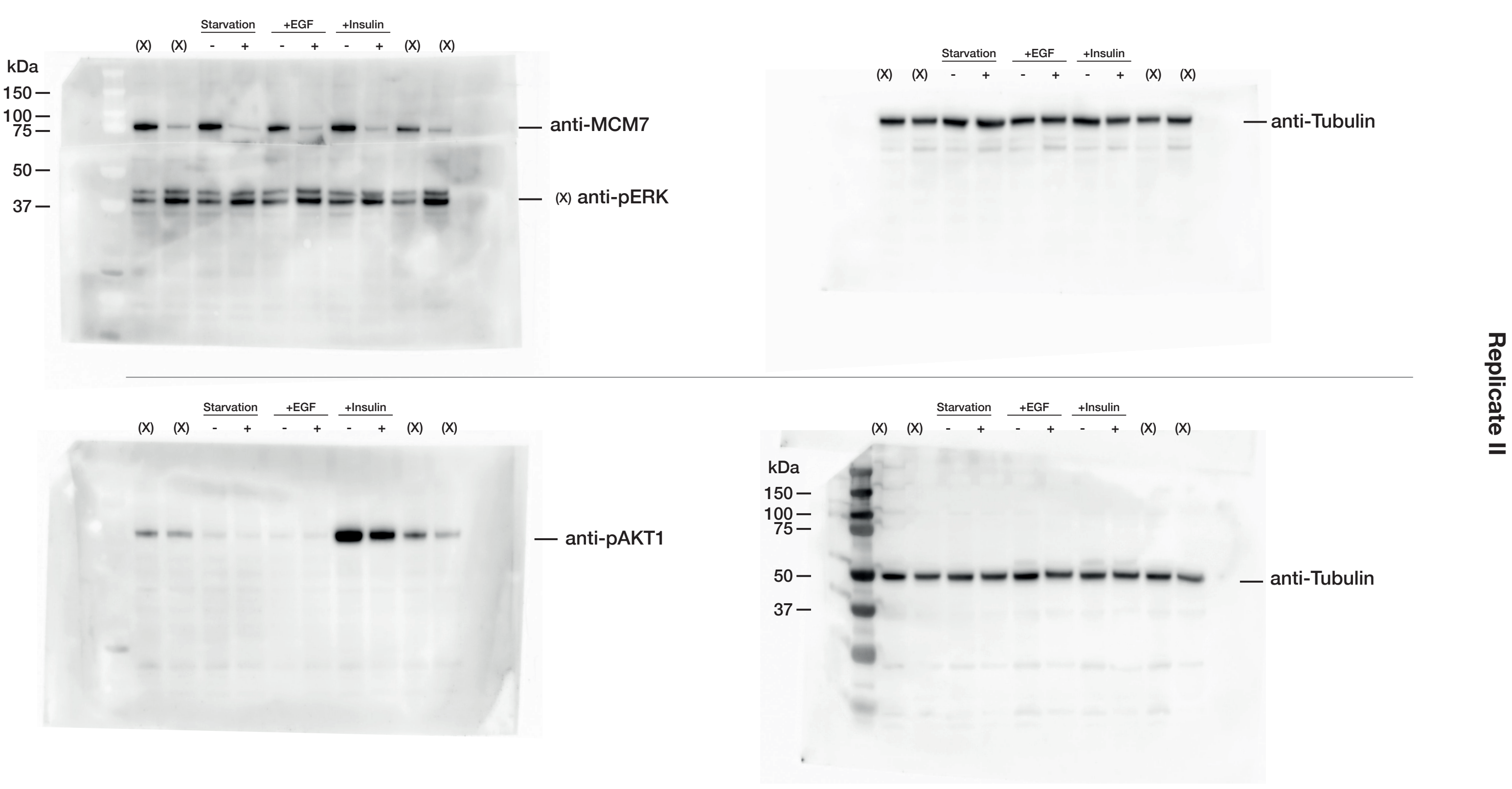

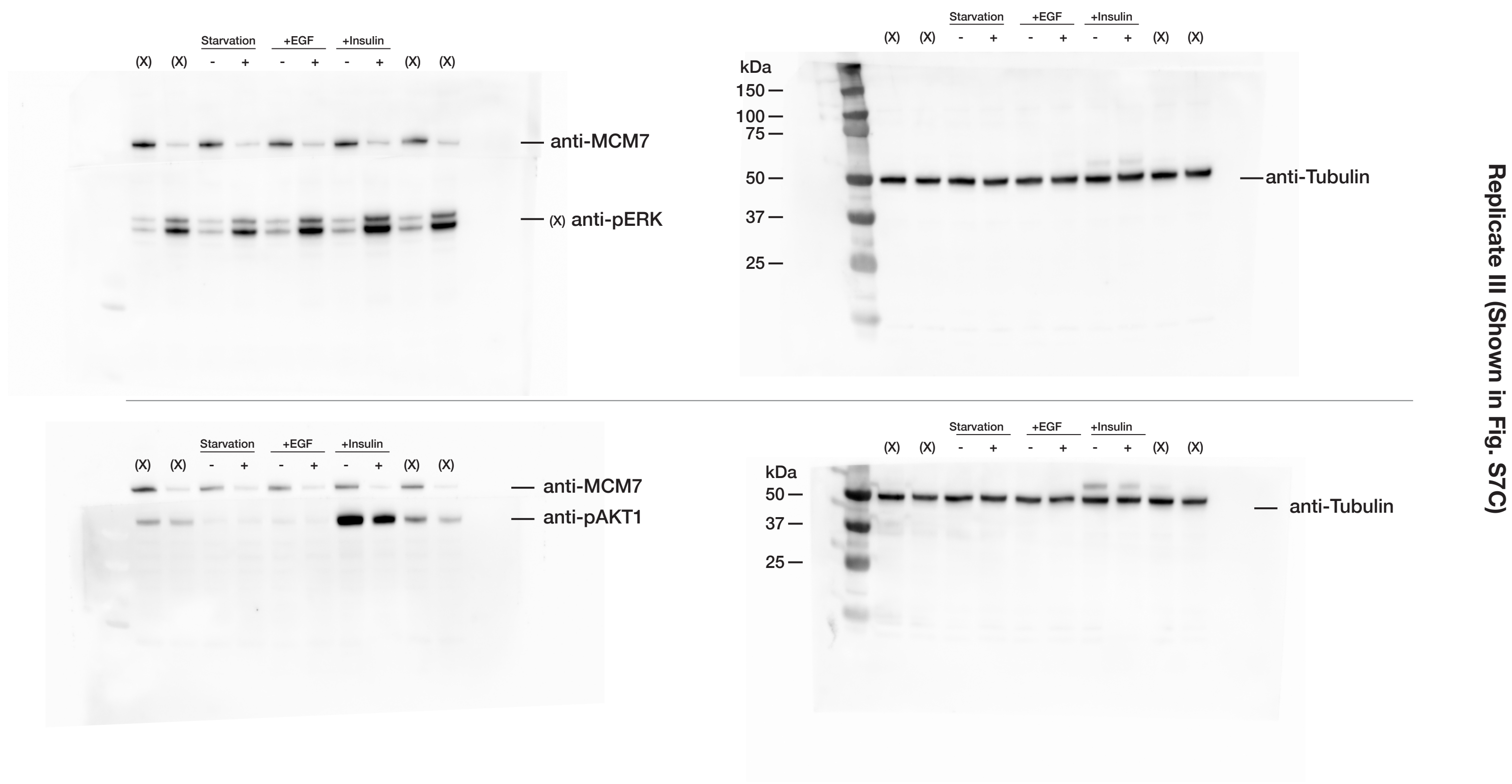

Original Westernblots Figure S7D&E

A549 shMCM7

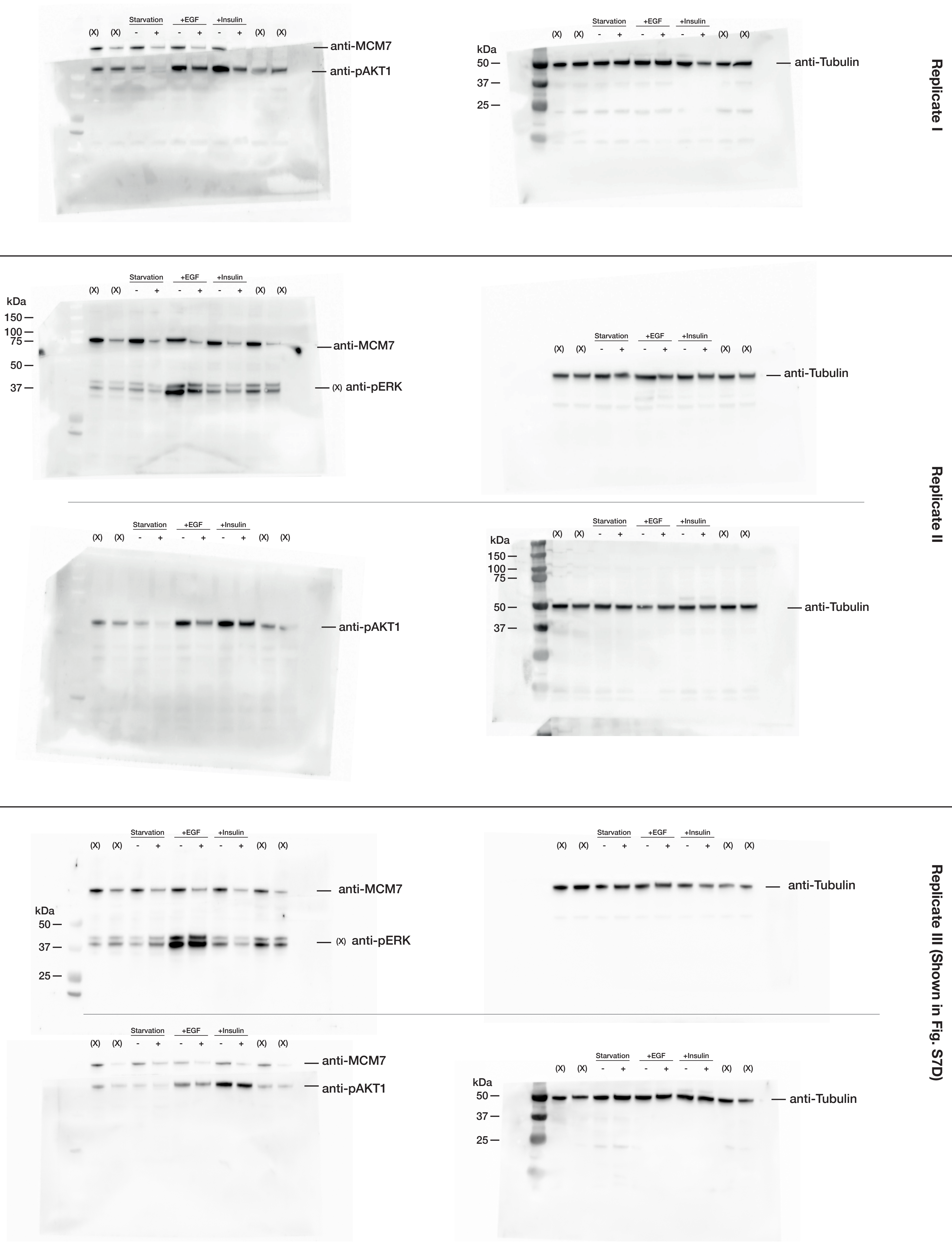

Original Westernblots Figure S7E

A375 Scramble

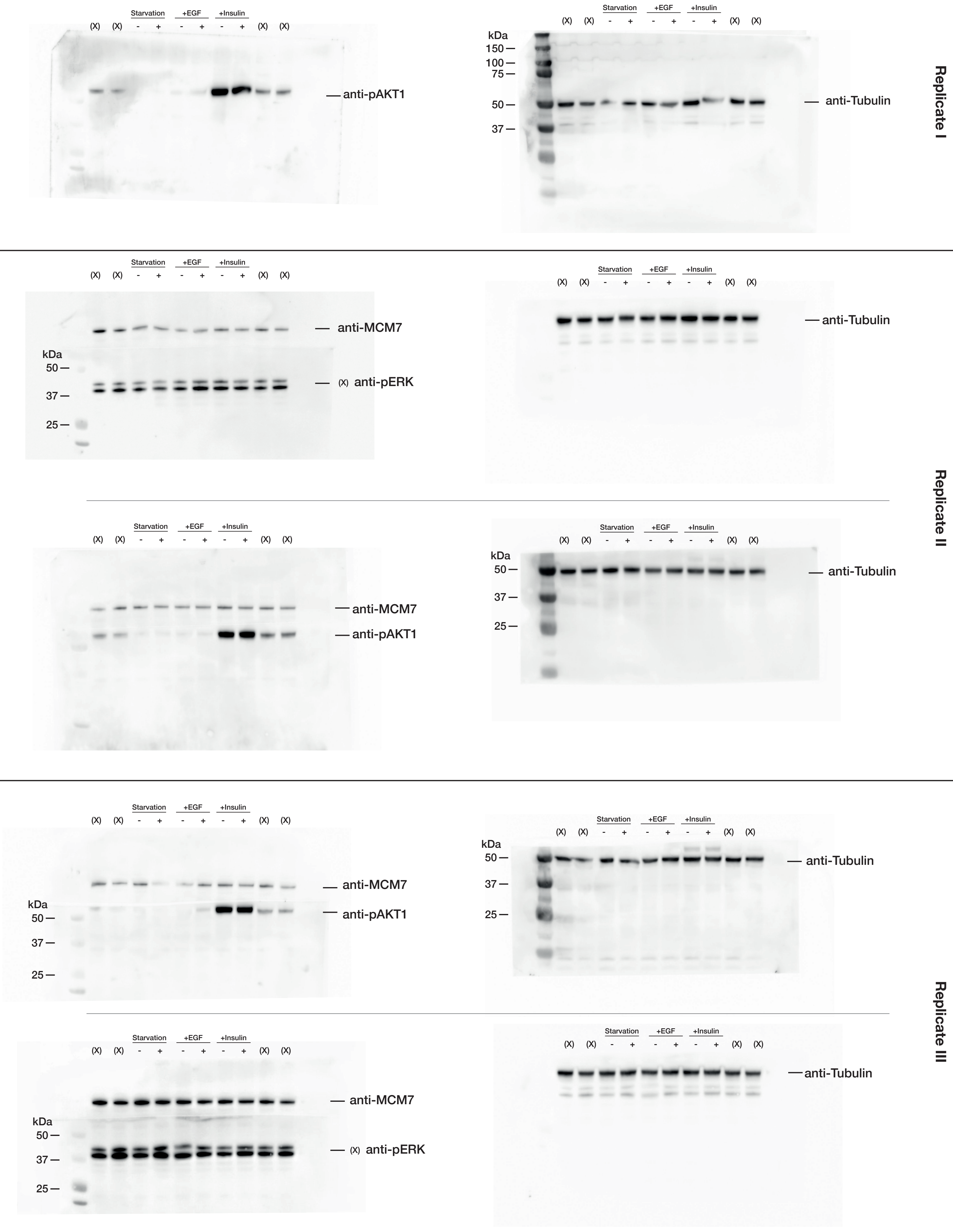

Original Westernblots Figure S7E

A549 Scramble

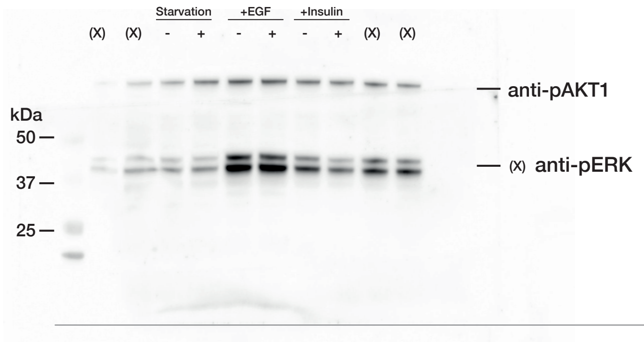

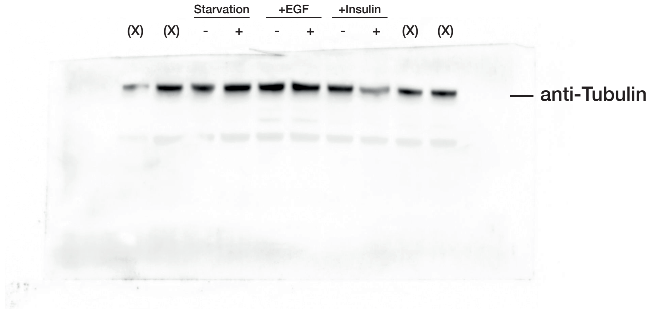

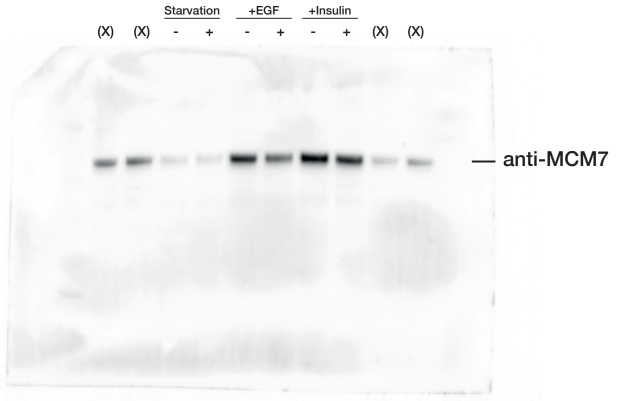

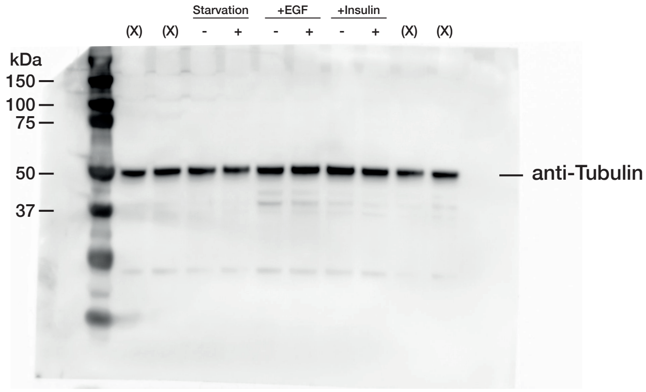

Replicate I

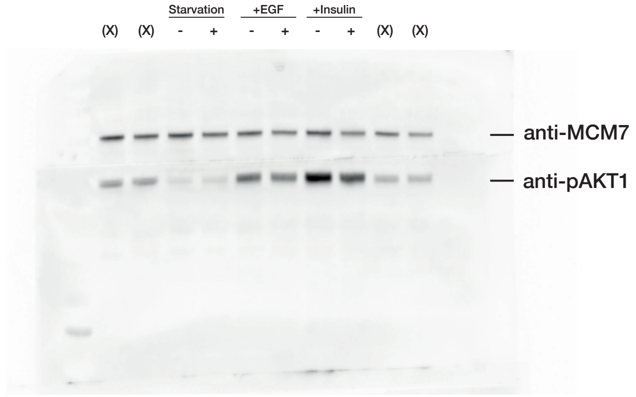

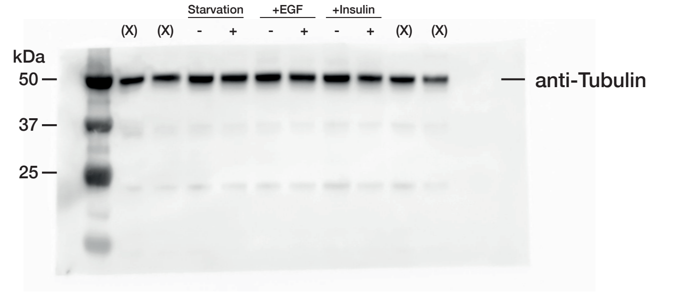

Replicate II

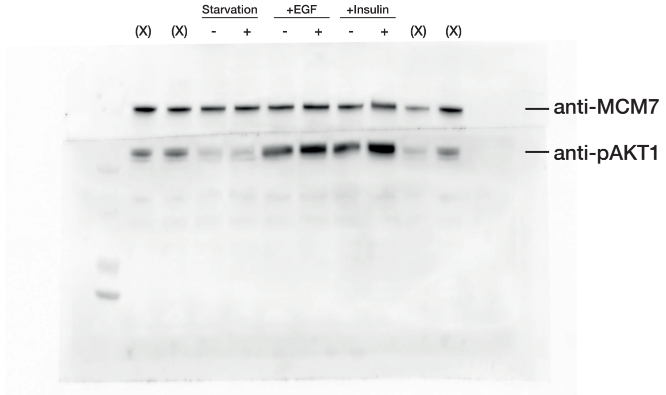

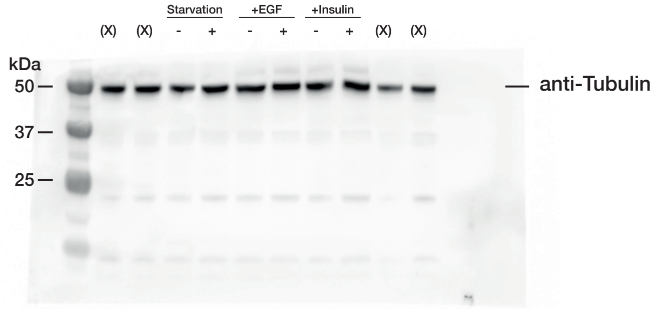

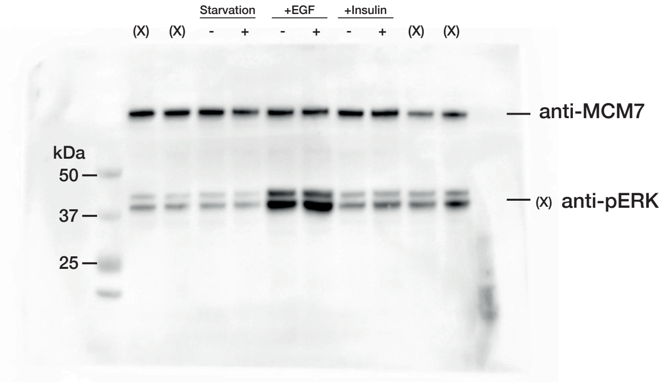

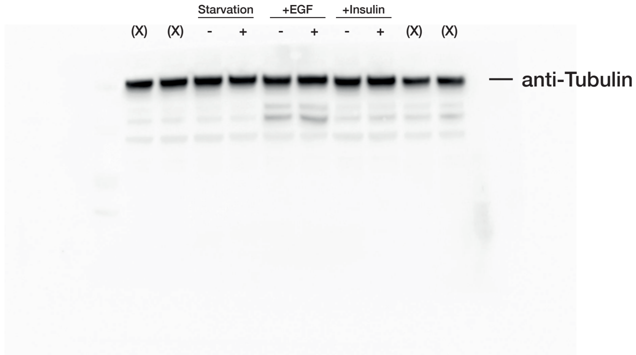

Replicate III
