# Supplementary material for: A DNA replication-independent function of pre-replication complex genes during cell invasion in C. elegans
Source: PLoS Biol. 2022 Feb 22;20(2):e3001317. doi: 10.1371/journal.pbio.3001317 (PMC8863262; doi:10.1371/journal.pbio.3001317)
Supplement: Attachment — Submitted filename: Point_by_point_response.pdf [file pbio.3001317.s020.pdf]

Point-by-point response

(*comments by the reviewers in italics*)

To reviewer1:
<u>Specific points:</u>
1. *One of the most important and interesting points of the paper is that the pre-Replication complex has a role in the post-mitotic AC to promote cell invasion. Because the lineage of the AC is complex and the timing of gene loss for most experiments in the work is not precise and likely before the AC is specified, this point needs to be clarified in the manuscript (esp., since the pre-RC has an established role in DNA replication associated with cell division). To reduce the function of mcm-7 and implicate a role in the post mitotic AC, the authors in part use a FLP/FRT-induced mosaic knockout system for mcm-7 that is induced via heat shock at the late L1/early L2 stage. One of the points the authors should state in this section of the results is that the AC is born in the early L2 stage (either Z1.ppp or Z4.aaa). Any mcm-7 knockdown before that time (such as possibly with the FLP/FRT system given the timing of induced knockout, and with whole body RNAi, which most of the work is done with) could implicate a role for mcm-7 in the mother cell that gave rise to the AC or in the cell division that gave rise to the AC rather than a role in the post-mitotic AC. This section should be organized to emphasize how the AC-specific RNAi establishes a role for mcm-7 in the post-mitotic AC itself (including describing specifically when the AC-specific strain becomes sensitive to RNAi) and not the ACs predecessor/mother cell. This point should also be emphasized in the following section with the control rescue of the mcm-7 knockout when mcm-7::cfp is driven by the lin-3 AC-specific promoter. To me this is the most important experiment in the paper, and I would have preferred an AC-specific degradation approach for MCM-7 (ZIF or AID), however, the lin-3 ACEL driven rescue is convincing, as long as early expression driven from this regulatory region is ruled out.*

Does MCM-7 act in the post mitotic AC or in its mother cell?
We agree, this is a central point that needed to be clarified- does MCM7 promote invasion in the post-mitotic AC or does it already act in the mother of the AC? As described in a recent publication by Attner et al . (ref 27), which performed precise lineage analysis of the AC and its precursors, the two cells with the potential to form the AC (Z1.ppp and Z4.aaa) are born during the third round of gonadal cell division in the mid to late L1 stage. For this reason, we applied the heat-shock to induce the FLPase in late L1/early L2 larvae, a time point **after** the two AC precursors had been born. Hence, inactivation of *mcm-7* after the birth of the AC does cause invasion defects. This is now explained in detail in the paragraph introducing the FLP/FRT-induced *mcm-7* mosaics (last para p.8 to 1st para p.9). Our conclusion is further supported by a detailed analysis of the timing of ACEL>MCM7::CFP expression in the rescue experiment (see below our response to point 2). What our data cannot distinguish is whether MCM-7 function is required during the L2 stage in the differentiating AC, e.g. to set up its invasive potential, or if MCM-7 acts later in the differentiated AC prior to BM breaching in L3. This point is now explicitly mentioned in the concluding paragraph (2nd para p.9).

2. The authors should confirm when mcm-7::CFP is first seen in the AC when driven by the AC-specific LIN-3 element—especially to make certain it is not expressed in the L1 stage in the gonad and cell that gives rise to the AC.

As recommended, we carefully evaluated the timing of ACEL>MCM-7 expression in 392 animals from hatching (early L1) to AC specification (mid L2), measuring in each animal the gonad length as a reference for the stage (see revised **suppl. Fig S4C**). This analysis clearly indicated that no ACEL>MCM-7 expression could be detected before the birth of the AC. Robust ACEL>MCM-7 expression was only detectable from the early L2 stage on in the differentiating AC. These data are in good agreement with the published data on the expression pattern of the ACEL enhancer (ref. 36).

3. The authors state, "Invasion by the post-mitotic AC is linked to the differentiation and proliferation of the underlying 1° VPCs that produce guidance signals, which attract the AC ventrally [7]. We therefore tested whether the AC invasion defects observed after down-regulation of pre-RC components are caused by a proliferation arrest of the VPCs." Notably, it has already been demonstrated that the AC can invade when the VPC divisions are blocked by Hydroxy Urea treatment. From Sherwood and Sternberg, Dev Cell 2003, "To determine if the AC invasion signal generated by the 1 vulval lineage is linked to cell division, cell division was blocked with HU, and animals were analyzed just prior to and after the normal time for AC invasion. While no invasion was observed in animals at the early-to-mid L3 (20/20 animals), at the mid-to-late L3, 95% of ACs attached to the undivided 1fated P6.p cells (19/20 animals; Figure 5E). " This original result should be cited and stated. It would be fine to test cell division with another method further in this paper, but it is important to cite the original finding

The results of the HU experiment by Sherwood et al. (ref. 7) is now mentioned in the result section (p.7 1st para). However, we think the *cki-1* overexpression experiment is important and should be included because CKI-1 over-expression arrests the 1° VPC in the G1 phase, the cell cycle stage when the pre-RC is active, rather than during S-phase after the initiation of DNA replication. This experiment is a clear indication that S-phase entry of the VPCs and initiation of DNA replication, licensed by the pre-RC, are not required for AC invasion (p.7 2st para).

4. How many animals were examined for the results shown in Figure 3A and 3B (cyd::GFP and RNR::GFP)? Can this be stated in the figure legend or text?

The numbers of animals scored are now mentioned in the legend to Figure 3A and 3B (p. 35).

5. I'm a little confused by Figure 4B. How many AC's were examined to make this graph? Can this be stated in the figure legend or graph?

The numbers of animals analyzed to produce the graph are shown in Figure 4B in brackets. This is now mentioned in the legend to Figure 4B (p. 37).

6. The authors state, "It should be noted that the gap the AC created in the BMs of psf-1(lf) mutants could not be expanded because the underlying VPCs had ceased to proliferate (Fig. 1A bottom row)" However, in Figure 1D when the authors block P6.p cell division by expressing cki-1 in the P6.p, the BM breach looks wide. ***Can the breach in these two conditions be quantified?*** *Perhaps the psf-1 does have an AC invasion defect?*

We have now measured the size of the BM gaps formed under the different conditions and found no significant differences. In *psf-1(lf)* mutants a fraction of the animals showed a smaller gap, presumably due to the absence of VPC division, but the average gap size in *psf-1(lf)* or *egl-17>cki-1::gfp* animals was not significantly different from the wild-type. This is now specifically mentioned for the case of *psf-1(lf)* (bottom of p.6). The results of the statistical analysis are as follows:

**Dunnett's multiple comparisons**	**Mean Diff.**	**95.00% CI of diff.**	**Summary**	**Adjusted P Value**	**A-?**	
control vs. *psf-1(ls)*	-0.9289	-2.545 to 0.6868	ns	0.3429	B	*psf-1(ls)*
control vs. *egl-17>cki-1::gfp*	0.7097	-1.268 to 2.687	ns	0.6495	C	*egl-17>cki-1::gfp*
**Test details**	**Mean 1**	**Mean 2**	**SE of diff.**	**n1**	**n2**	**q**
control vs. *psf-1(ls)*	6.758	7.687	0.7134	46	29	1.302
control vs. *egl-17>cki-1::gfp*	6.758	6.049	0.8733	46	16	0.8127

7. The work in melanoma cell lines examining the replication-independent functions of MCM7, by analyzing gene expression in cells arrested at the G1/S boundary after knockdown of MCM7 is interesting. While a role for MCM7 in promoting the expression of the PI3K came from this and was confirmed in C. elegans, where reciprocal findings also found? Specifically, did the work in melanoma cells also identify a role for MCM7 in promoting MMP expression as it does in the AC? It looks like this work might have found the opposite—" GO enrichment analysis of the molecular functions and biological processes of these core MCM7 target genes indicated that many of the up-regulated genes encode extracellular matrix, integrin, actin and ß-catenin interacting proteins". Can you comment/compare on the expression of MMPs in cancer cell lines and your analysis. This is helpful for the field, as pointing out differences as well as similarities is important in building a complete picture of this important and complex problem—pre-RC and cell invasion in different contexts.

This is an interesting point. As suggested, we specifically examined the RNAseq data of the A375 and A549 cell lines for significant changes in MMP expression and observed several differentially expressed MMPs. Though, there was no consistent pattern between the two cell lines. One interesting candidate gene that was significantly up-regulated in both lines is TIMP2, which encodes an MMP inhibitor. These results are now specifically mentioned in the result section (last para p.13 to 1st para p.14).

8. ***Does PI3K signaling loss on its own result in an AC invasion defect?*** *It is important that the authors perform AC-specific RNAi on age-1 and aap-1 to establish the requirements of PI3K signaling in the AC on its own. Many aspects of cell biological processes are redundant or provide robustness. This will provide broader insight into the role of the pre-RC into cell invasion, even if age-1 and aap-1 loss on their own don't result in an invasion defect.*

As suggested, we tested if inactivation of PI3K signaling affects AC invasion. For this purpose, we examined homozygous F2 *age-1(mg44)* loss-of-function mutants to eliminate the maternal contribution (see new ref 44). We observed in 23% of age-1(lf) mutants defects in BM breaching -an AC was present in all *mg44* animals as they exhibited normal vulval induction. These new data are now described in the result section (p.15, 2nd para) and shown in the new **suppl. Fig S10**.

9. Discussion section: "The PI3K pathway may be activated in the AC by the INA-2/PAT-2 ab-integrin complex on the invasive membrane [6]" It should be INA-1/PAT-3. There is no INA-2/PAT-2 integrin.

Apologies for this sloppy error! The sentence at the bottom of p.16 now reads: "The PI3K pathway may be activated in the AC by the **INA-1/PAT-3** $\alpha\beta$-integrin complex on the invasive membrane."

To reviewer 2:

<u>Specific points:</u>
1. The title and abstract of the current submission imply that the pre-RC, as a complex, participates in the transcriptional regulation of AC invasion. However, some pre-RC components that are essential for replication licensing do not seem to play a role in invasion, and the paper in its present form does not provide evidence that the genes identified in the screen act within the pre-RC complex. The reported observations could imply that some members of pre-RC play independent roles as parts of other molecular machines, a concept that already has a precedent (see #2 for references) as several members of the pre-RC complexes were reported to participate in cellular multi-protein complexes other than pre-RC, mediating a variety of cellular functions. This point should at least be discussed.

Are all pre-RC components required for AC invasion?
This is a valid comment: Our data cannot distinguish whether all or just a subset of the pre-RC genes are required for AC invasion. Even though the MCM proteins are strongly conserved in metazoans and act as a single protein complex, we only observed a phenotype for *mcm-7* RNAi (**Tab. 1**). However, we observed invasion defects after RNAi of *orc* genes as well as for *cdc-6* and *ctd-1*, which are all recruited before the MCM2-7 into the pre-RC. This suggests that the entire pre-RC or most of its components regulate AC invasion. To account for the possibility that only some of the pre-RC genes are required for AC invasion, we have changed the revised manuscript in numerous places, starting with the title, abstract, text on p. 6, 1st para, and we explicitly mention the possibility that the pre-RC genes we have identified as regulators of AC invasion may act outside of the canonical pre-RC on p.18, 1st para.

2. Precedents for interactions of pre-RC components with other multi-protein complexes that play a role in regulating cellular functions have been established (for example, for Drosophila ORC6 PMID: 12878722; centromeric association of ORC2, PMID: 15215892; for CDT1 at kinetochores PMID: 22581055 and PMID: 30154187). Appropriate citations should be included.

These references have been included and the alternative functions of pre-RC genes in other protein complexes are now discussed on p.18, 1st para and additional refs 64-68.

3. The expression patterns of MCM7 analyzed in the paper are interesting and support the conclusions that this protein plays a role in invasion. It would be interesting to follow the expression of other pre-RC components in a similar manner.

We have now included the expression pattern of *cdt-1*, which, similar to *mcm-7*, is expressed in the AC nucleus before and during BM breaching. CDT-1::GFP expression is now shown in the new **suppl. FigS2** and discussed on p.8 1st para. Besides MCM-7 and CDT-1, we also observed CDC-6 and ORC-2 reporter expression in the AC, but their expression patterns were less robust and we therefore chose not to include these data in the manuscript.

4. MCM-7 was the only component of the MCM helicase required for AC invasion, and the conclusion that the helicase activity of pre-RC plays a role in the regulation of AC invasion is based on the phenotype of MCM-7 variants harboring ATPase mutations. Although the ATPase activity of MCM-7 is essential for pre-RC helicase activity, the results observed in the current study do not rule out an independent and distinct role for MCM7 ATPase activity that is unrelated to its role in facilitating the MCM2-7 helicase. Because AAA ATPases often manifest more than a single function in cellular activity, this possibility should be discussed.

It is possible that the ATP binding domain of MCM-7 may be required for the function of an alternative complex that contains MCM-7 but not the other MCMs to provide helicase activity. Even though we deem this scenario as rather unlikely, we mention this alternative explanation on p.10 2nd para and in the discussion on page 16,1st para.
